# Distribution and length of muscle spindles and their 3D visualisation in the medial gastrocnemius of male and female rats

**DOI:** 10.1111/joa.13895

**Published:** 2023-05-25

**Authors:** M. Piotr, K. Skieresz‐Szewczyk, H. Jackowiak, J. Celichowski

**Affiliations:** ^1^ Department of Neurobiology Poznan University of Physical Education Poznan Poland; ^2^ Department of Histology and Embryology Poznan University of Life Sciences Poznan Poland

**Keywords:** 3D reconstruction, dimorphism, morphology, skeletal muscle

## Abstract

The spatial distribution of the medial gastrocnemius muscle spindles of 10 male and 10 female rats was analysed under a light microscope, and for the first time, visualised using a 3D model of the muscle. Serial cross‐sections of the medial gastrocnemius muscles were separated into 10 divisions along with the proximo‐distal axis. The muscle spindles of the rat medial gastrocnemius were predominantly distributed on the proximo‐medial divisions of the muscle. There were no sex‐related differences in the distribution of the studied receptors. A single division contained an average of 2.71 receptors for animals of both sexes. Moreover, the calculated lengths of male and female rat muscle spindles were comparable, and average lengths did not significantly differ (3.30 ± 1.47 mm for male and 3.26 ± 1.32 mm for female rats). Therefore, the present results fill gaps in recent observations concerning similarities in muscle spindle numbers between male and female animals, despite significant differences in muscle mass and size.

## Graphical abstract

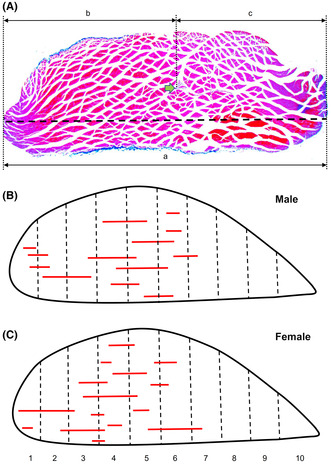

## 1 INTRODUCTION

Muscle spindles are crucial for the control of body position and movement. The distribution of these spindles has been investigated in numerous studies, which have indicated that the distribution was not homogeneous and differed among various mammalian muscles (Barker & Chin, 1960; Bredman et al., 1991; Eldred et al., 1998; Kokkorogiannis, 2004; Ovalle et al., 1999; Scott & Young, 1987). Regarding the sheep multifidus muscle, James et al. (2022) confirmed that the majority of muscle spindles were distributed in the middle portions of the muscle belly. In a similar study, Lian et al. (2022) measured the relative distance from muscle spindles to nerve entry points for four limb muscles of C57BL/6 female mice, and a 2D distribution of the muscle spindles was presented for a sample muscle. In addition, the heterogenic distribution of muscle spindles was shown for the chosen cross‐sections (Lian et al., 2022). Zeller‐Plumhoff et al. (2017) documented that muscle spindles in the solei of male mice were concentrated in the middle portion of these muscles and were presented using a one‐axis layout. A recent study identified considerable differences in the density of gastrocnemius muscle spindles between male and female rats, with a 39% higher muscle mass shown in males compared to females (Gartych et al., 2021). However, there were no sex differences in the number of muscle spindles, the size of the muscle spindles or the number and diameter of the intrafusal muscle fibres. The spatial distribution of muscle spindles, as well as their lengths, have not been analysed in this study. Although the body mass, central nervous system and muscle mass are considerably greater in male rats than in females (Gartych et al., 2021), no studies have been identified which compared sex‐related differences in the distribution of these receptors in hindlimb muscles. Currently, 3D visualisation is not used in neuroanatomy to illustrate the distribution of spindles in the muscle, and the distribution of these structures has only been demonstrated on a 2D plane or a single axis.

The mass, size and architecture of a skeletal muscle differ considerably depending on sex. The extrafusal muscle fibres of the medial gastrocnemius muscle have a greater diameter in male rats than in female rats (Mierzejewska‐Krzyżowska et al., 2014). Furthermore, male rats have a larger pennation angle of the medial gastrocnemius than that of females (34.6° vs. 27.3°, respectively) (Manal et al., 2006; Takahashi et al., 2022). Additionally, Chow et al. (2000) found that the length of muscle fibres was greater in male individuals than in females. However, the determination of whether these differences in muscle architecture are related to the length of the muscle spindles (measured as the length of intrafusal fibres) has not been documented, although literature data indicate that the length of muscle spindles for different muscles of certain species varies considerably, with a range of 0.14 to 5.40 mm (Barker & Chin, 1960; Eldred et al., 1998; Kierner et al., 1999; May et al., 2018). This study aimed to present and compare the distribution and length of muscle spindles in the medial gastrocnemius of male and female rats. For this purpose, the 3D reconstruction technique was used for the first time to graphically present the muscle spindle distribution. The obtained results will contribute to the knowledge concerning sex differences in the sensory innervation of skeletal muscles.

## 2 MATERIALS AND METHODS

The animal characteristics, muscle preparation method and staining procedure for standard histological transverse cross‐sections along with the long axis of 10 male and 10 female rat medial gastrocnemius muscles described by Gartych et al. (2021) were used in this study.

**FIGURE 1:**
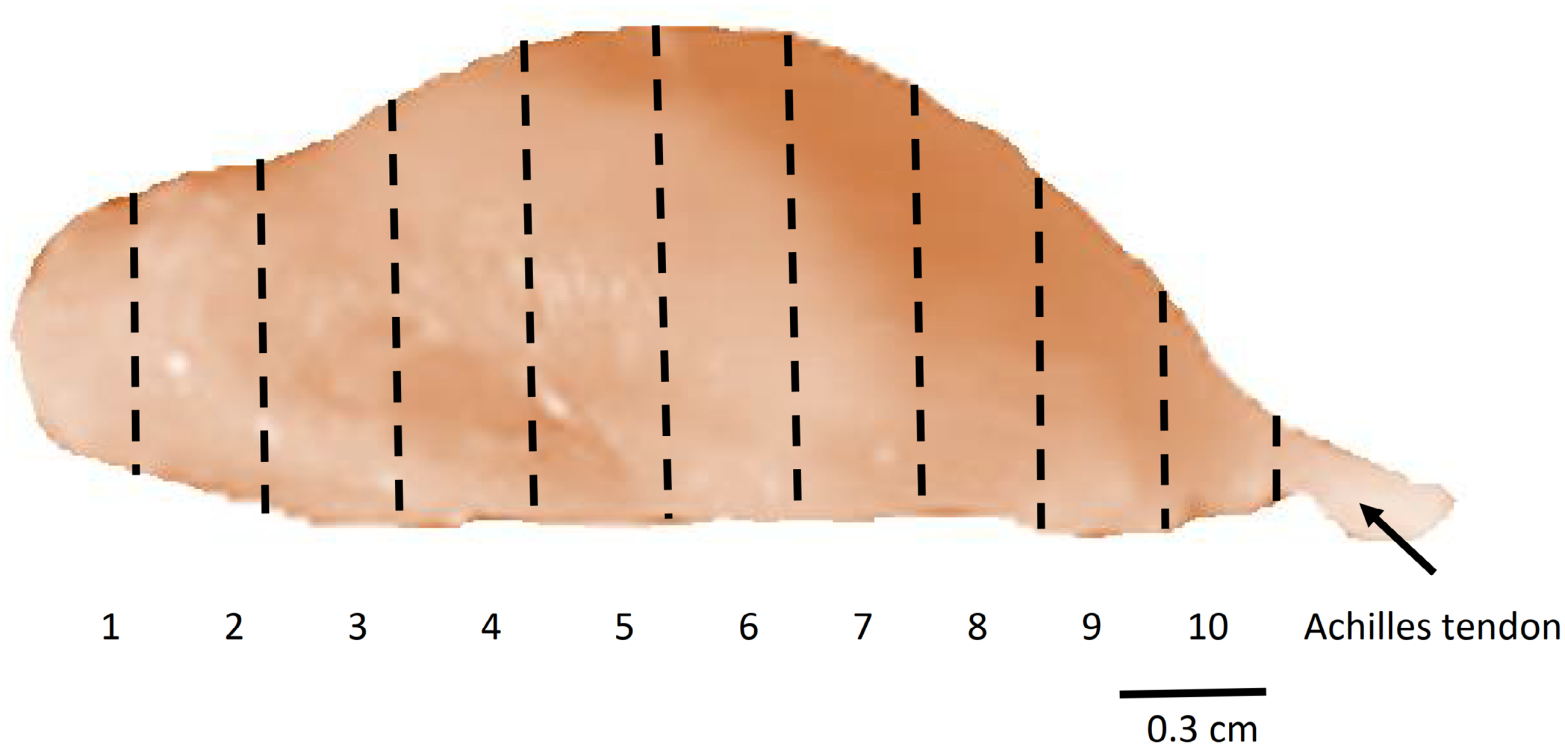
Macrophotograph of the fixed right medial gastrocnemius muscle of a male rat (outer surface) and distribution of 10 divisions along with the proximo‐distal axis.

The number of muscle cross‐sections used was determined and the muscle was then divided into 10 proportional divisions, numbered 1–10, along with the proximo‐distal axis (Figure 1). One division included 170–260 cross‐sections in males and 130–260 cross‐sections in females. The presence of muscle spindles on the serial muscle cross‐sections was determined using a light microscope (Prolab, Poland). The beginning and end of each muscle spindle were marked and all cross‐sections were proportionally distributed into 10 divisions. The length of the muscle spindle (the length of the longest intrafusal muscle fibre) was determined, and the distribution of individual muscle spindles along with the 10 divisions of the medial gastrocnemius muscle of male and female rats was obtained. Additionally, the medio‐lateral position of each spindle within the muscle was determined. Magnifications of 10× (0.38 μm/pixel) and 63× (0.04 μm/pixel) were used for the muscle spindle photographs.

To perform 3D reconstruction and observation of one entire male medial gastrocnemius muscle with muscle spindles, serial cross‐sections were observed and photographed in a single line under a magnification of ×1.25 using an Axioskop 2 Plus light microscope (Zeiss) with a Progress Gryphax Microscope Camera (Jenoptik). The 1934 images were separated into 10 divisions with an average of 200 images for each. The following steps were used to create the 3D reconstruction: (1) conversion to a single channel (green channel), (2) alignment, (3) recalculation into uniform coordinates (necessary for the next step—segmentation), (4) segmentation, (5) resampling in the X, Y and Z directions (the resampling was necessary to reduce the resolution of the stack to speed up the calculation of the surface; additionally, resampling in the Z‐direction smoothens the surface image) and (6) surface generation using the professional computer software, Amira (Thermo Fisher Scientific, ver. 2020) (Movie 1). Based on the 3D reconstruction, an animation showing the 3D structure of the muscle and muscle spindles in the Z and Y axes was prepared using the Animation Director tool in the Amira software. In this 3D reconstruction, the muscle is transparent and the muscle spindles are highlighted in green.

Statistical analysis was performed using Statistica v.13.0 software (TIBCO Software Inc.). Quantitative parameters were studied and data were plotted on graphs using the mean and standard deviation (SD), which were grouped by sex and muscle section. The Shapiro–Wilk test was used to test the normality of the distribution, and Levene's test was used to test the homogeneity of the variance. To analyse the sex differences for variables that met the condition of normality and homogeneity of variance, a Student's *t*‐test (*t*) was performed. In situations where at least one variable of the parameter pair did not meet the condition of normality or homogeneity of variance, the *U*‐Mann–Whitney test was used (*Z*). An analysis of variance for multivariate systems (*F*) was used to examine length differences of muscle spindles between muscle parts in males and those in females. Significance was set at *p* < 0.05.

## 3 RESULTS

**FIGURE 2:**
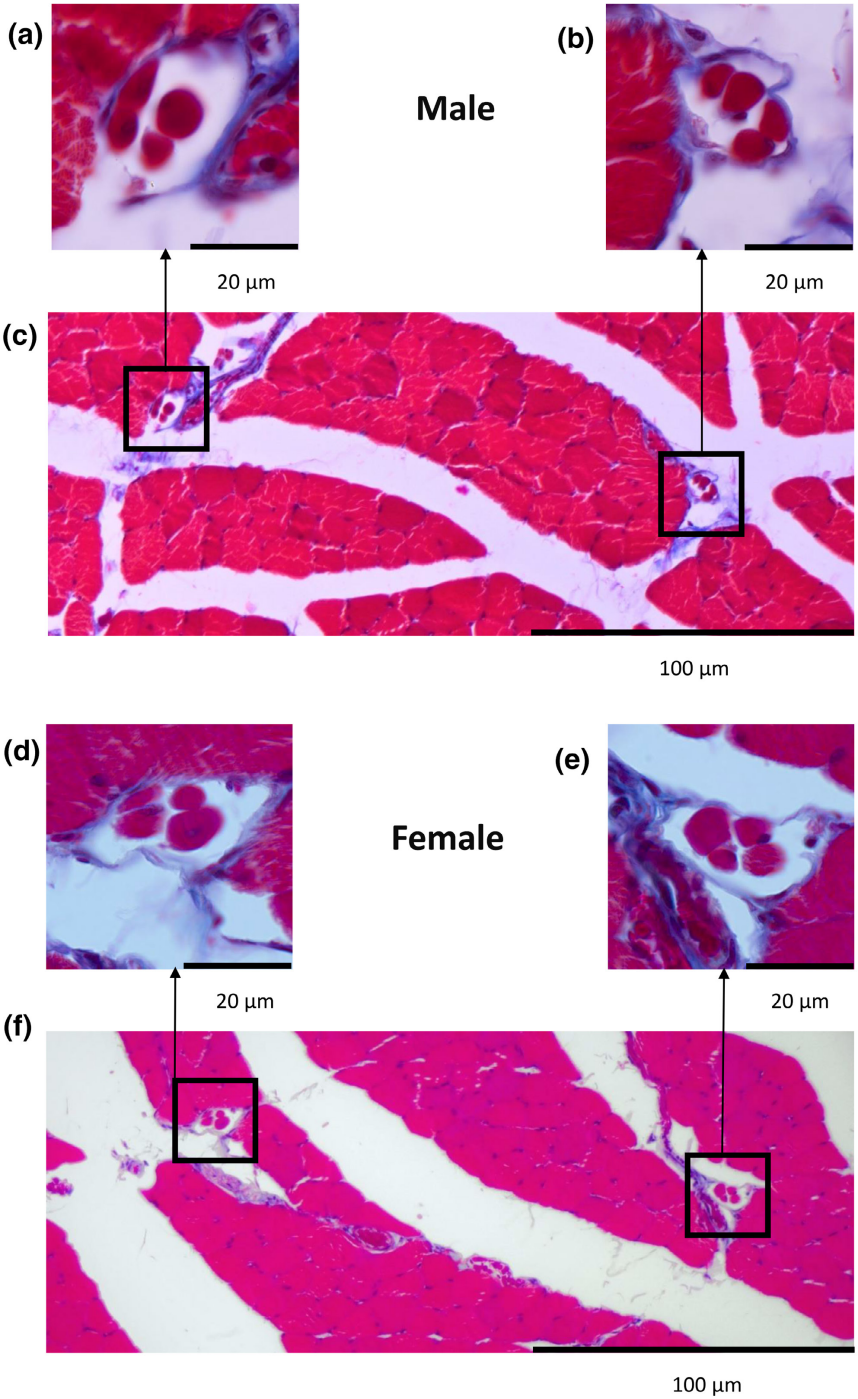
Microphotographs of male (upper panel—a–c) and female (lower panel—d–f) cross‐sections of the middle part of the medial gastrocnemius muscle. For each of the two presented muscle fragments, the higher magnification of two muscle spindles are visible.

The 10 analysed medial gastrocnemius muscles revealed 148 muscle spindles for males and 152 for females. The cross‐sections of the muscle spindles showing intrafusal muscles surrounded by capsules in male and female medial gastrocnemius muscles are presented in Figure 2.

**FIGURE 3:**
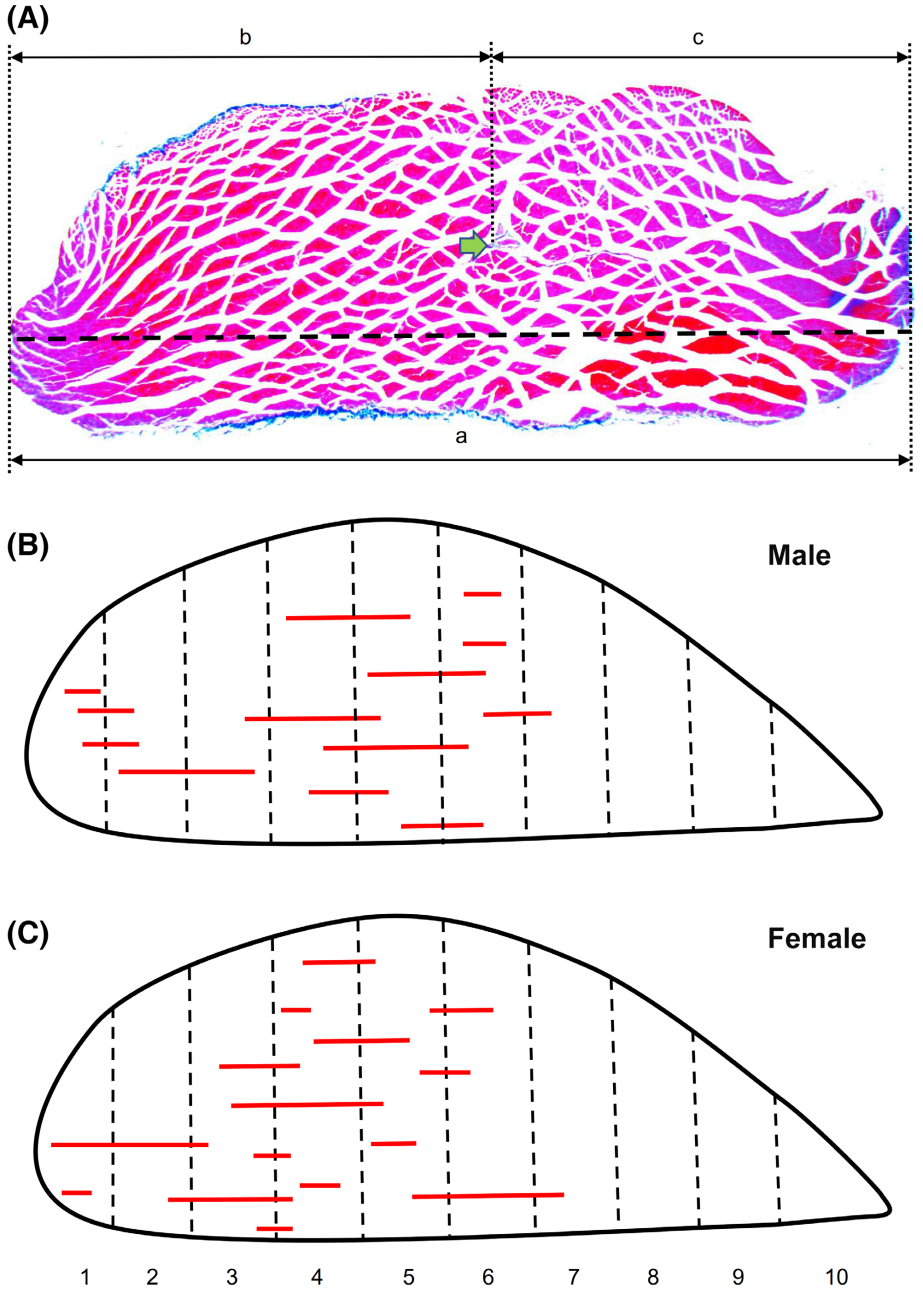
Distribution of muscle spindles on the horizontal sectional plane of the rat medial gastrocnemius. (A) Photograph of a sample cross‐section of the female medial gastrocnemius with one muscle spindle indicated by the green arrow, which illustrates the method of positioning the studied receptor. The position of each muscle spindle on the muscle width axis (a) was determined using the distance to the borders (b, c). Additionally, the position of each muscle spindle along with the muscle and the distribution within 10 divisions was determined basing on number of muscle cross‐sections showing visible muscle spindles. (B, C) Distribution of 13 and 15 muscle spindles on a horizontal division plane for a left male and left female muscle, respectively. The scheme assumes that muscle spindles are located along with the longitudinal axis of the muscle.

**FIGURE 4:**
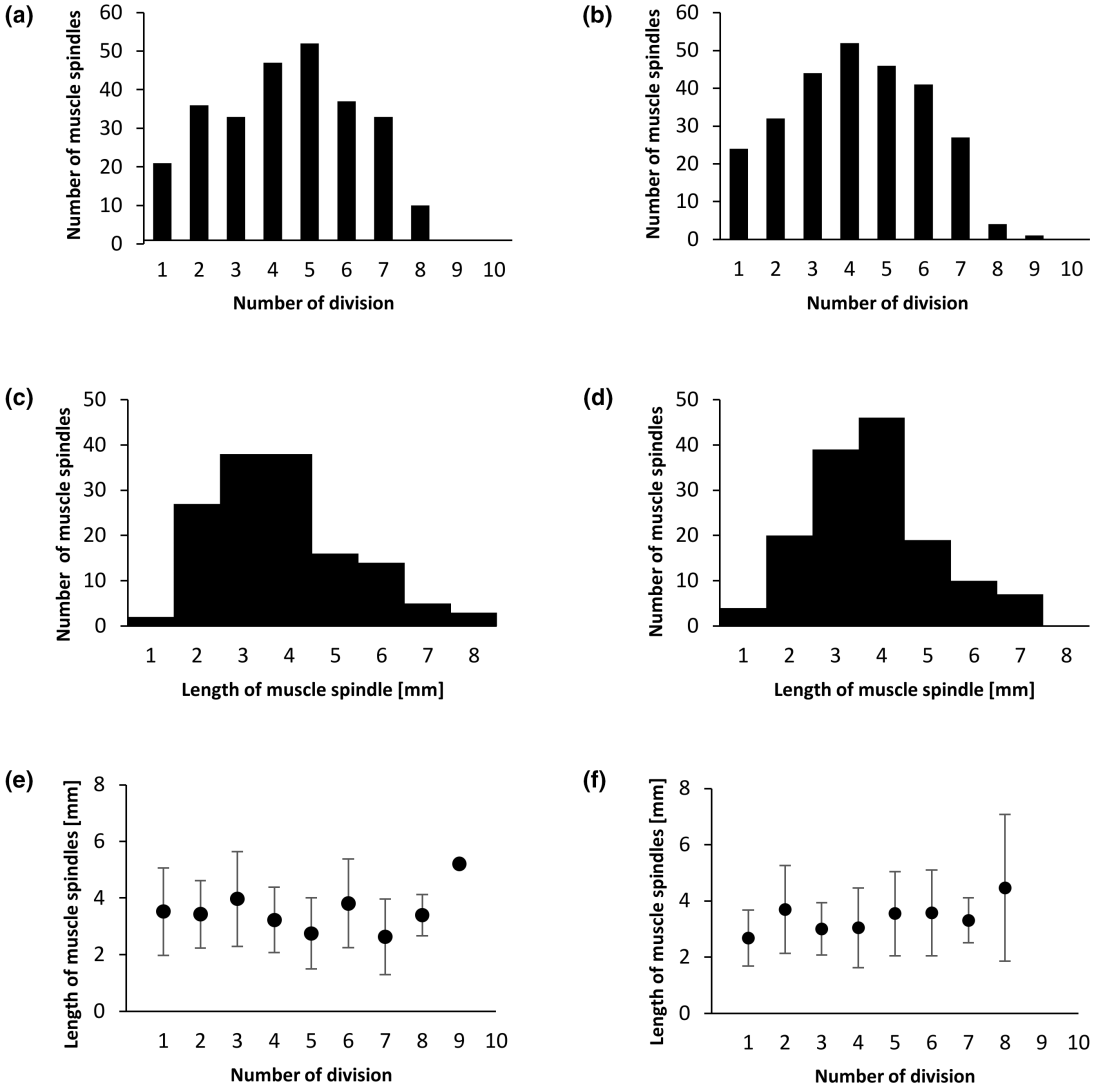
(a–f) Distribution and length of muscle spindles in a male (left) and female (right) medial gastrocnemius of a rat. (a, b) The number of muscle spindles located within 10 designated divisions. (c, d) The lengths of muscle spindles in the studied muscle. (e, f) The mean values ±SD of the length of muscle spindles belonging to all divisions. The figure presents collected data for muscle spindles from 10 male and 10 female rats.

A schematic localisation of the muscle spindles of the two studied muscles (one male and one female, as seen from the antero‐posterior projection plan) is shown in Figure 3. The muscle spindles were predominantly distributed in the widest part of the muscle, which was located in divisions 4–6 for both male and female specimens (Figures 3 and 4a,b, Movies 2 and 3). The proximal part of the medial gastrocnemius muscles was characterised by a higher number of muscle spindles than those in the most distal part of the muscle, where divisions 9 and 10 lacked these spindles (Figures 3 and 4a,b, Movies 2 and 3). The distribution along with the proximo‐distal axis of the muscle for males and females was normal and spindles were located along with the longitudinal axis of the medial gastrocnemius muscle (Figure 3, Movies 2 and 3). The average number of muscle spindles per division amounted to 2.71 ± 1.81 for males and 2.71 ± 1.95 for females (the difference was non‐significant: *t* = 0.00; *p* = 1.00). Moreover, the distribution of muscle spindles for all successive divisions did not differ when data for male and female muscles were compared (*p* > 0.05 for all divisions). The average location of the muscle spindles along with the muscle was calculated based on the distribution of spindles in all serial divisions and was determined as the 5th division (the average values were 4.40 for males and 4.18 for females).

The lengths of the spindles were similar for male and female muscles (0.78–7.41 mm in males and 0.68–6.54 mm in females; Figure 4c,d), and the mean lengths amounted to 3.30 ± 1.47 mm for male rats and 3.26 ± 1.32 mm for female rats (the difference was non‐significant, *Z* = 0.05; *p* = 0.96).

Figure 4e,f presents the length of muscle spindles in 10 muscle divisions. For this analysis, the middle point of each muscle spindle was assigned to the respective division. The plot presents the mean values and standard deviations of muscle spindle lengths belonging to the serial divisions (the length of muscle spindle in serial divisions as well as sex differences in the distribution were non‐significant, *F* (8,283) = 1.90; *p* = 0.059).

## 4 DISCUSSION

Recently, it was demonstrated that there is a lack of sex‐related differences in the number of muscle spindles in the rat medial gastrocnemius, despite a significant difference in muscle mass (Gartych et al., 2021). In the present paper, we verified that there were no differences in the distribution or length of muscle spindles between male and female rats, despite the evident differences in muscle mass and size. These observations broaden the knowledge of sex differences in skeletal muscle structure and innervation.

Muscle spindles have been studied in numerous muscles with variable functions and structure and considerable variability in their distribution has been reported. For example, in spinal stabilisers, multifidus muscles from merino sheep muscle spindles were shown to occur predominantly in the central part of this muscle (James et al., 2022). In contrast, human digastric muscle spindles were present in the anterior belly (Saverino et al., 2014), whereas in the quadrilateral masseter of male rabbits, muscle spindles occurred near the middle and anterior parts of the muscle, and there was a descending trend in the localisation of the spindles towards the insertion of the muscle (Bredman et al., 1991). Relatively consistent observations have been noted regarding hindlimb muscles. Barker and Chin (1960) found that the majority of muscle spindles were located in the proximal half of two limb muscles of cats, the rectus femoris (part of the quadriceps muscle responsible for knee extension/hip flexion) and the fifth interosseus (responsible for finger adduction and abduction). Similarly, studies of these receptors in cat hindlimb muscles have indicated that in the peroneus tertius (crucial for eversion of the foot) and peroneus longus (plantar flexor of the foot and recurrent foot), muscle spindles were located mainly in proximal regions (Scott & Young, 1987). The current results for the rat medial gastrocnemius confirmed a similar distribution pattern of muscle spindles to that found in cat limb muscles (i.e. the majority of spindles were located in the proximo‐medial parts). However, a similar concentration of muscle spindles in the proximal parts of hindlimb muscles was not reported in the locomotor soleus muscles of male C57BL/6 mice. In this case, the muscle spindles were observed in the belly of the muscle and covered approximately 20%–90% of the entire muscle length (Zeller‐Plumhoff et al., 2017).

Muscle spindle length was similar for male and female rats. This absence of differences in muscles spindle length and those of recently reported muscle spindle diameters and intrafusal fibres (Gartych et al., 2021) contrast with the considerable intersex differences in extrafusal muscle fibre diameter, muscle mass and size, which have been documented for the same muscle (Mierzejewska‐Krzyżowska et al., 2011), along with differences in extrafusal muscle fibre length (Chow et al., 2000). These observations indicate that factors affecting differences in muscle mass, such as the activity of male hormones during development (Bell, 2018; Gegenhuber et al., 2022), have limited influence on the development of muscle spindles in skeletal muscles. A possible reason for this may be a protective role of the collagen capsule surrounding the muscle spindle (Kennedy & Yoon, 1979) or the fact that muscle spindle maturation occurs earlier than that of extrafusal muscle fibres, which has been noted for human masseter and biceps brachii muscles (Österlund et al., 2011).

The reported length of muscle spindles in the rat medial gastrocnemius (average length of 3.30 and 3.26 mm for male and female muscles, respectively) in this study can be compared to those in the literature for various mammalian muscles. The length of intrafusal muscle fibres in different mammalian muscle spindles (predominantly in human and cat muscles) can vary between 8 and 10 mm (Boyd, 1962). The literature indicates that this parameter appears to depend on body and/or muscle size. For example, muscle spindle lengths for small human muscle tensor tympani (damping the noise produced by chewing) amount to 1.49 mm, but those for the smallest human stapedius muscles (damping the vibrations of the stapes) are only 0.49 mm (Kierner et al., 1999). Moreover, the length of the muscle spindles varied from 1.5 to 4 mm in both men and women in the longus coli (neck flexor) and multifidus muscles (spine stabiliser) (Boyd‐Clark et al., 2002). In hindlimb muscles of female C57BL/6 mice, the muscle spindle was shorter than of the rat muscles studied here and varied in the tibialis anterior (dorsiflexion of the foot and its inversion) and extensor digitorum longus (dorsiflexion of the foot) by approximately 0–2 mm, whereas the locomotor gastrocnemius and soleus muscles showed 0–1.5 mm of variation (Lian et al., 2022).

This study used the technique of 3D visualisation to observe the muscle spindles in sample medial gastrocnemius muscle. This was the first study to reveal the distribution of the muscle spindles and their spatial layout in a three‐dimensional virtual manner since previous studies have only prepared 2D visualisations or models constituted in a single axis (Lian et al., 2022; Zeller‐Plumhoff et al., 2017).

## 5 CONCLUSION

The muscle spindles in rat medial gastrocnemius were distributed predominantly in the proximo‐medial part of the muscle, and both the distribution of muscle spindles and their length in male and female animals were similar despite significant differences in muscle mass and size. The 3D reconstruction technique, which was used for the first time here in the study of the spatial distribution of muscle spindles, can be considered a useful tool for further studies assessing the sensory innervation of skeletal muscles.

## AUTHOR CONTRIBUTIONS

J. Celichowski provided the conception of the study; M. Piotr, K. Skieresz‐Szewczyk, H. Jackowiak and J. Celichowski acquired the data; M. Piotr, K. Skieresz‐Szewczyk and H. Jackowiak involved in the analysis of the data; M. Piotr drafted the manuscript; H. Jackowiak and J. Celichowski critically revised the manuscript; M. Piotr, K. Skieresz‐Szewczyk, H. Jackowiak and J. Celichowski gave the approval for the article.

## Supporting information


Movie 1

Movie 2

Movie 3

## Data Availability

Data from this study are available from the corresponding author upon reasonable request.
